# Synthesis and Characterization of a Novel Chitosan-Based Nanoparticle–Hydrogel Composite System Promising for Skin Wound Drug Delivery

**DOI:** 10.3390/md22090428

**Published:** 2024-09-21

**Authors:** Yueying Huang, Shuting Hao, Jiayu Chen, Mengyuan Wang, Ziheng Lin, Yanan Liu

**Affiliations:** 1 Department of Food Science and Engineering, Ningbo University, Ningbo 315800, China; huangyueyingnbu@163.com (Y.H.); 17857395807@163.com (S.H.); 18857475416@163.com (J.C.); w17858875621@163.com (M.W.); anrimoce@outlook.com (Z.L.); 2 Ningbo Key Laboratory of Detection, Control and Early Warning of Key Hazardous Materials in Food, Ningbo Academy of Product and Food Quality Inspection, Ningbo Fibre Inspection Institute, Ningbo 315048, China; 3 College of Food Science and Technology, Nanjing Agricultural University, Nanjing 210095, China

**Keywords:** nisin, nanoparticles, thermo-sensitive hydrogels, composite drug delivery system, bacteriostasis

## Abstract

As a natural preservative, nisin is widely used in the food industry, while its application in biomedicine is limited due to its susceptibility to interference from external conditions. In this study, a nanoparticle–hydrogel composite system was designed to encapsulate and release nisin. Nisin nanoparticles were identified with a smooth, spherical visual morphology, particle size of 122.72 ± 4.88 nm, polydispersity coefficient of 0.473 ± 0.063, and zeta potential of 23.89 ± 0.37 mV. Based on the sample state and critical properties, three temperature-sensitive hydrogels based on chitosan were ultimately chosen with a rapid gelation time of 112 s, outstanding reticular structure, and optimal swelling ratio of 239.05 ± 7.15%. The composite system exhibited the same antibacterial properties as nisin, demonstrated by the composite system’s inhibition zone diameter of 17.06 ± 0.83 mm, compared to 20.20 ± 0.58 mm for nisin, which was attributed to the prolonged release effect of the hydrogel at the appropriate temperature. The composite system also demonstrated good biocompatibility and safety, making it suitable for application as short-term wound dressings in biomedicine due to its low hemolysis rate of less than 2%. In summary, our nanoparticle-based hydrogel composite system offers a novel application form of nisin while ensuring its stability, thereby deepening and broadening the employment of nisin.

## 1. Introduction

Nisin is the only bacteriocin in the world that is permitted as a food additive, owing to its GRAS (Generally Regarded as Safe) status approved by the WHO and FDA [1,2]. Nisin, one of the metabolites of *Lactococcus lactis*, is a natural antimicrobial with broad-spectrum antibacterial activity, which is commonly employed in food preservation [3]. In the process of its extensive application, some characteristics of nisin that hinder its effectiveness have gradually emerged, including susceptibility to external environment interference leading to loss of antibacterial activity, susceptibility to hydrolysis by proteolytic enzymes, and poor stability under certain pH conditions [4,5]. Furthermore, nisin has been reported to play a role in immunological modulation, but due to the aforementioned characteristics, little information is available regarding its application in biomedicine [6,7]. Many efforts have been made to ensure the stability of nisin and extend its application. Many methods have been developed to modify and encapsulate it, such as hybridization with active substances or grafting of active groups [8,9], conjugation with polymers [1,10], preparation of encapsulated sustained-release systems [6,11,12], etc.

Nisin is often utilized in the food sector as films or nanofibers for incorporation into food packaging [13,14]. Although nisin currently has fewer biomedical applications than food applications, existing data show that it is mostly utilized in biological dressings and drug delivery systems [15]. At present, there is significant interest in the encapsulation and delivery system of active substances as a research topic, primarily due to its advantages of local drug delivery, ensuring the activity of substances, and high safety [16,17]. The inhibitory mechanism of nisin in destroying the bacterial cell wall demonstrates that moderate contact with the bacteria is essential to suppress its activity, which means that a delivery system capable of stabilizing and releasing it as needed is required [6]. In addition, biomedical applications necessitate delivery mechanisms with high safety and biocompatibility. The application of nanotechnology has achieved remarkable success in enhancing the antibacterial property and stability of nisin as well as the ability to achieve prolonged release, which has been widely used in the food industry [18,19]. However, its use in biomedicine is currently limited due to a scarcity of closed carriers that can make contact with humans. Another typical application is the direct use of nisin to produce hydrogels, which has stringent criteria for the many components involved [20,21]. All elements that influence the activity of nisin must be considered while designing the product. Thus, the nanoparticle–hydrogel composite system is an appropriate form of nisin for biomedical purposes. To date, many studies have been carried out on nanoparticle–hydrogel composite systems, but the encapsulation of nisin in this system has not been documented [22,23,24].

Chitosan (CS), a product derived from the deacetylation of chitin from marine arthropods, is a natural cationic polymer with good degradability and biocompatibility and is widely used in delivery systems such as hydrogels and nanoparticles [25]. γ-Polyglutamic acid (γ-PGA) is a natural polymer with excellent hydrophilicity, biocompatibility and safety. Due to the presence of carboxyl groups, the negatively charged γ-PGA is able to provide multiple cation-binding sites in aqueous solution, a phenomenon that has been used in nanoencapsulation, self-assembled hydrogels, and other applications [26,27]. There are many cases of synthesizing nanoparticle drug delivery systems using ionic interactions between polymer CS and γ-PGA, mainly relying on the interaction between -NH4^+^ in CS and -COO^-^ in γ-PGA, which leads to spontaneous cross-linking to form nanoparticles [28,29]. In addition to this, CS is able to cross-link with other substances to form hydrogel encapsulation systems. Nisin Z, one of the variants of nisin, is an amphiphilic small molecule peptide with strong inhibitory effects on Gram-positive bacteria, and has also been reported to inhibit certain Gram-negative bacteria [30,31,32]. It has been reported that nisin Z can be extracted from a wide range of lactic acid bacteria strains, e.g., wild strains, strains isolated from marine organisms, etc. [33,34]. Nisin Z also has a wide range of applications, and is capable of assisting aquatic organisms in resisting pathogenic bacteria, in addition to food preservation and preservation [35,36]. Nisin Z is positively charged in aqueous solution and thus is able to bind to negatively charged γ-PGA via electrostatic interactions, while -COO^-^ on the surface of the γ-PGA is able to further cross-link with CS to form nanoparticles [37]. The nanoparticles are positively charged by chitosan encapsulation, and can be loaded onto supportive hydrogels in order to realize multi-environmental applications. Nanoparticles synthesized using nisin Z and pectin, as reported previously, exhibited a low PDI and high potential value, indicating a strong interaction [38]. However, CS can only be solubilized in an acidic environment, which have been reported to also promote the dissolution and encapsulation of nisin Z [18,39,40]. Therefore, we selected CS as the main constituent of the composite material.

In this study, we used a thermo-sensitive hydrogel based on CS to encapsulate nanoparticles (NPs) made of CS, γ-PGA, and nisin Z. Following this approach, we created an encapsulating, sustained-release, and biocompatible nanoparticle–hydrogel composite system. The surface properties of the nanoparticles are important factors in the formation of composite systems [41]. Particle characteristic parameters, such as the particle size, potential, and polydispersity coefficient, reflected the appropriate particle volume and agglomeration degree of nanoparticles. SEM was used to examine the appearance and conditions of the particles, corresponding to the particle properties. Accordingly, a series of characterizations were performed to assess the properties of the NPs. In addition, the composition of the hydrogel was also optimized to make the hydrogel more compatible with the NPs. The groups with better performance were comprehensively selected as carriers according to a series of characterization results, particularly microstructure, sustained release effect, and swelling performance. This study focused on the antimicrobial properties and biocompatibility of the composite system, which will be evaluated and predicted for applications in biomedicine and other fields.

## 2. Results and Discussion

### 2.1. Characterization of Nanoparticles

#### 2.1.1. Properties of Particles

**Figure 1:**
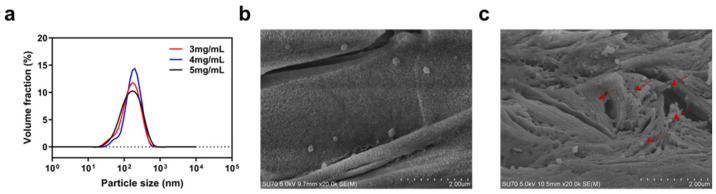
Properties of particles. (**a**) the particle size of nanoparticles with nisin Z concentration of 3, 4, and 5 mg/mL in the final solution, (**b**) SEM of NPs, (**c**) SEM of NPs in freeze-dried nanoparticle–hydrogel composite system. The NPs that were loaded on the hydrogel are indicated by the red arrows in the figure. Scale bar: 2 μm.

NPs with concentrations of 3, 4, and 5 mg/mL of nisin Z in the final NPs solution were prepared to determine the particle characteristics. As seen in Figure 1a, the nanoparticle sizes at the three concentrations of nisin Z were concentrated between 100 and 200 nm, with nanoparticles with concentrations of 3 and 4 mg/mL of nisin Z showing a small amount of concentrated distribution between 10 and 100 nm. As shown in Table S1, the distribution of nanoparticles with nisin Z concentrations of 5 mg/mL was more uniform for minimum PDI; therefore, it was selected as the loaded particles for the composite system. The average hydrodynamic diameter of nanoparticles with nisin Z concentrations of 5 mg/mL was 122.72 ± 4.88 nm (mean ± SD) and the PDI was 0.473 ± 0.063, demonstrating a minor state of aggregation. The obtained zeta potential of 23.89 ± 0.37 mV indicates that the surface of the nanoparticles was positively charged and had a certain level of stability. The surface conditions of the NPs were observed and photographed using SEM. Figure 1b shows the smooth surface and spherical shape of the NPs. As shown in Figure 1c, the shape of the NPs changed slightly after being loaded into the hydrogel, showing signs of extrusion or entanglement. In summary, our NPs have good surface properties and particle properties, providing an excellent basis for the formation of composites, though further design and optimization are still needed for both NPs and hydrogels to maintain the same excellent properties.

#### 2.1.2. Entrapment Efficiency of Nisin Z

Previous reports have shown that the encapsulation efficiency of nisin Z in nanoparticles gradually decreases with an increasing nisin Z concentration. The measured encapsulation efficiency of nanoparticles with nisin Z concentrations of 3, 4, and 5 mg/mL was approximately 80%, and it increased with the increase of nisin Z concentration in our experiment. The encapsulation efficiency of the nanoparticles containing 5 mg/mL nisin Z was 86.18%, indicating a good encapsulation effect. The reason for this phenomenon in this experiment is related to the acidic environment to which nisin Z is exposed. It has been reported that the solubility of nisin Z is related to the pH [39]. Studies have shown that the solubility of nisin Z is higher under acidic conditions, and the corresponding encapsulation efficiency is also higher [18,40]. In the preparation of nanoparticles, the solution is acidic due to the presence of acetic acid, with a pH of approximately 3.40, thus providing suitable environmental conditions for the maximum encapsulation of nisin Z. The encapsulation efficiency of a previously reported nisin Z nanoparticle for beef preservation was 86.6%, demonstrating an excellent preservation effect [42]. In terms of the encapsulation effect, our nanoparticles remain at the same gradient as those in previous studies, which is critical for subsequent activation. The loading capacity of NPs with 5 mg/mL of nisin Z in the final solution was 59.35%, and the loading capacity of NPs with 3 mg/mL and 4 mg/mL of nisin Z in the final solution was 37.16% and 44.08%, respectively. The degree of superiority in the loading capacity results for each NP sample is consistent with the encapsulation rate. Accordingly, the weight percentage of other substances in each NPs can be analyzed. The mass percentage of CS and γ-PGA in the NPs with a concentration of 5 mg/mL of nisin Z in the final solution was 40.65%. In the NPs with 3 mg/mL and 4 mg/mL of nisin Z in the final solution, it was 62.84% and 55.92%, respectively.

### 2.2. Characterization of Hydrogel and Composite

#### 2.2.1. Choosing the Best Formula

**Figure 2:**
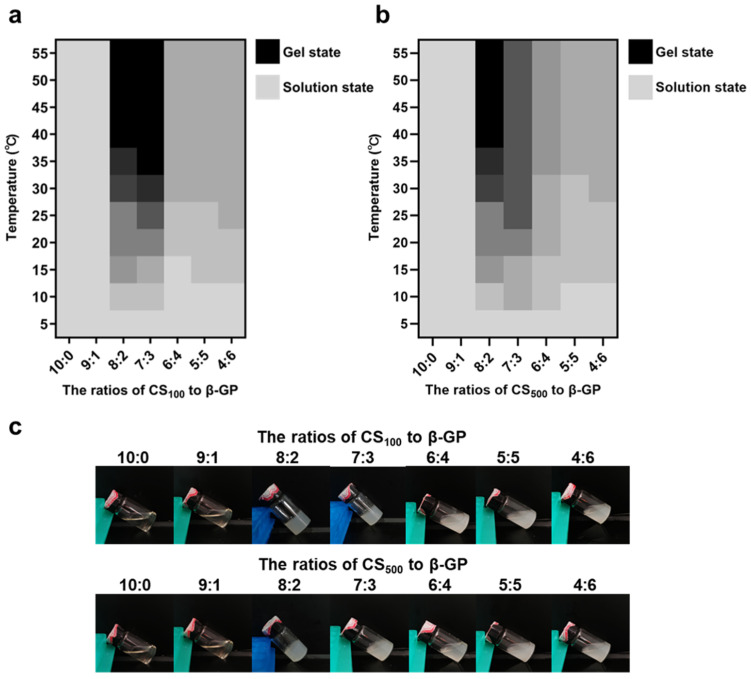
Schematic of optimal formulation selection. (**a**) gel condition of each sample of CS_100_ as a function of temperature, (**b**) gel condition of each sample of CS_500_ as a function of temperature, (**c**) schematic representation of the gel state of each sample.

In this study, the effects of time, temperature, and raw material ratio were investigated to select better hydrogels. We also compared the effects of two groups of CS with different molecular weights. Figure 2 shows the state change and final state diagram of each group of samples as the temperature rises from 5 °C to 55 °C. The color (from gray to black) represents the gradual weakening of the fluidity of the samples; that is, gray represents the solution state, and black represents the gel state without fluidity. The two types of CS with different molecular weights showed similarities in terms of the influence of the raw material ratio. When the content of β-Glycerol phosphate disodium salt (β-GP) was low, the fluidity did not change with the increase in temperature. When the β-GP content was high, the fluidity of the sample decreased slightly with an increase in temperature and then remained stable, but no gel change occurred. When the ratios of CS with molecular weight of 100 kDa (CS_100_) to β-GP were 8:2 and 7:3, the temperature-sensitive gel phenomenon occurred; the same occurred when the ratio of CS with molecular weight of 500 kDa (CS_500_) to β-GP was 8:2. According to the data on the time required for gelation at 37 °C, the above three groups completed gelation within 5 min; additionally, the time required for gelation gradually decreased with the increase in temperature. Similarly, studies have investigated the effect of CS and β-GP concentrations on gelation, with the results showing that when the concentration of β-GP was 3% (*w*/*v*), the gelation phenomenon occurred within 0.5–1% (*w*/*v*) of CS concentrations. This result is consistent with our study, and gelation of CS and β-GP can only occur within the appropriate dosage range [43]. Three groups of samples (Gel_100-73_, Gel_100-82_, and Gel_500-82_) capable of temperature-sensitive gelation were preliminarily investigated, and subsequent studies were conducted on the basis of these samples to further compare the performance differences.

#### 2.2.2. Rheological Characterization

**Figure 3:**
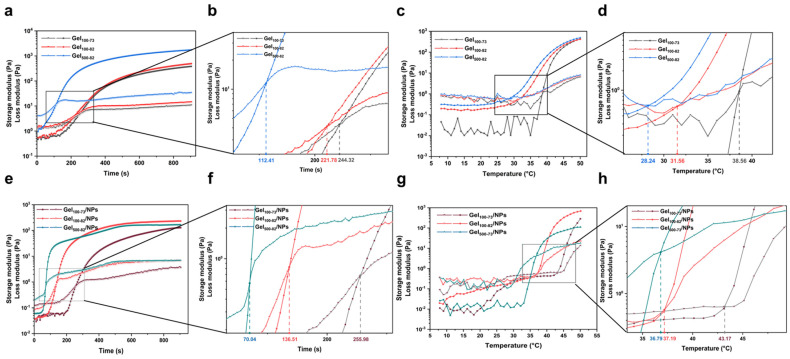
Storage modulus (G′) and loss modulus (G″) of the samples. (**a**) time scanning of gel samples, (**b**) detail map of time scanning of gel samples, (**c**) temperature scanning of gel samples, (**d**) detail map of temperature scanning of gel samples, (**e**) time scanning of composite samples, (**f**) detail map of time scanning of composite samples, (**g**) temperature scanning of composite samples, (**h**) detail map of temperature scanning of composite samples.

The time and temperature of gelation were redetermined through time and temperature scanning using rheology. The storage modulus and loss modulus exhibited variations with the passage of time or increase in temperature, while the point of intersection between these two curves indicated the respective gelation time and gelation temperature (Figure 3). In the time-scanning process, Gel_500-82_ first underwent gelation for approximately 1 min and 52 s, and its storage modulus reached a high, stable value within 5 min of gelation, relating to the gel strength. The gelation times of Gel_100-73_ and Gel_100-82_ were relatively close, as can be seen from the temporal scan detail map; Gel_100-73_ gelation occurred at approximately 4 min and 5 s, and Gel_100-82_ gelation occurred at approximately 3 min and 42 s (Figure 3b). Similar to the results of previous experiments, all of our gel samples were able to undergo gelation within a short time [44]. Gel_500-82_ was also the first to undergo gelation transition at approximately 28.24 °C in the temperature scanning process. The gelation temperature point of Gel_100-82_ was 31.56 °C, while that of Gel_100-73_ was 38.56 °C (Figure 3d). Under the influence of temperature change, the modulus change curve of the sample shows a small fluctuation, likely associated with the environment or equipment, especially for Gel_100-73_. Small fluctuations had little effect on the measurement of the experimental results, but they were sufficient to reflect the significant influence of temperature change on the sample state, further verifying the temperature responsiveness of the hydrogel. The results obtained from the rheological experiments were consistent with those obtained when the gel effect was investigated. Additionally, our samples exhibited a more sensitive temperature response with the same gelation time as previous hydrogels of the same type [44,45]. The rapid gelation of the above samples at approximately the physiological temperature of the human body (37 °C) provides a prerequisite for their wide application in biomedicine. The time scans of each composite (Figure 3e,f) showed that the addition of NPs did not have significant effect on the gelation time, and each sample was still able to gel within 5 min. However, due to the more complex composition, the gel temperature of the composites in Figure 3g,h showed an increase, and the temperature scanning curves showed small fluctuations. The gel temperature of Gel_100_-_73_/NPs even exceeded 40 °C, which is still considered to be suitable for biological excipients.

#### 2.2.3. FTIR Characterization

**Figure 4:**
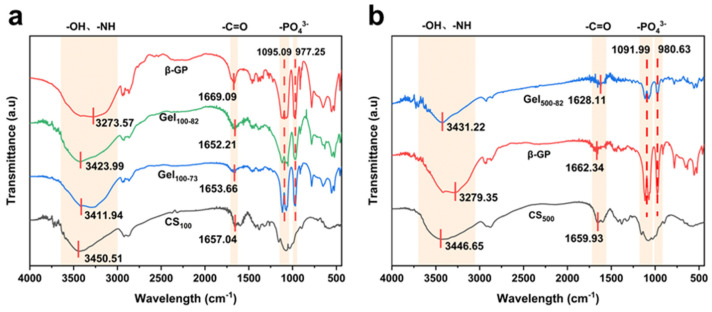
FTIR spectrogram of samples of the CS_100_ progenitor (**a**) and the CS_500_ progenitor (**b**).

The gel formation mechanism can be clearly observed in the FTIR characteristic curve. The FTIR results obtained were identical to those obtained in previous hydrogel experiments [44,46]. The stretching of -OH and -NH corresponded to the strong and wide overlapping peak at 3450 cm^−1^ in the infrared spectrum of CS_100_, and the band at 1657 cm^−1^ was attributed to the bending vibration of -C=O (Figure 4a). The peak of the infrared spectral line of CS_500_ was similar to that of CS_100_ (Figure 4b). For β-GP, the peak of stretching -OH was found at 3273 cm^−1^. The asymmetric stretching vibration of -PO_4_^3−^ appeared at 1095 cm^−1^, and the symmetrical stretching vibration peak of -PO_4_^3−^ was observed at 977 cm^−1^. The overlapping peaks of stretching -OH and -NH can also be seen on the bands of gel samples. However, the electrostatic interaction between -PO_4_^3−^ and -NH caused the peak shape and corresponding wavelength of the peak tip to change slightly. The characteristic peaks of -PO_4_^3−^ on the bands of the gel samples coincided with those of β-GP. Moreover, the small magnitude of the shift exhibited by the peak of -C=O may be caused by the formation of hydrogen bonds during gelation.

#### 2.2.4. Microstructure

**Figure 5:**
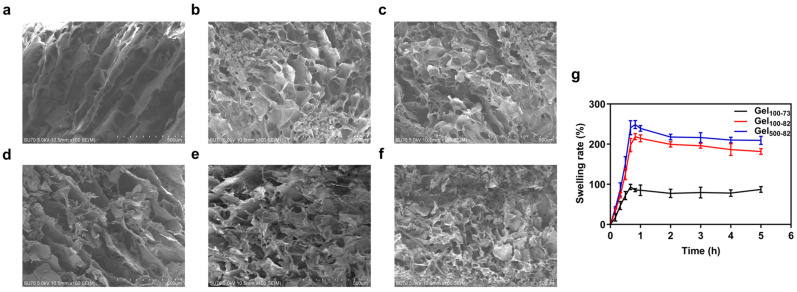
SEM of Gel_100-73_ (**a**), Gel_100-82_ (**b**), and Gel_500-82_ (**c**) SEM of Gel_100-73_/NPs (**d**) Gel_100-82_/NPs (**e**) and Gel_500-82_/NPs (**f**) and swelling rate of gel samples (**g**). Scale bar: 500 μm.

**Figure 6:**
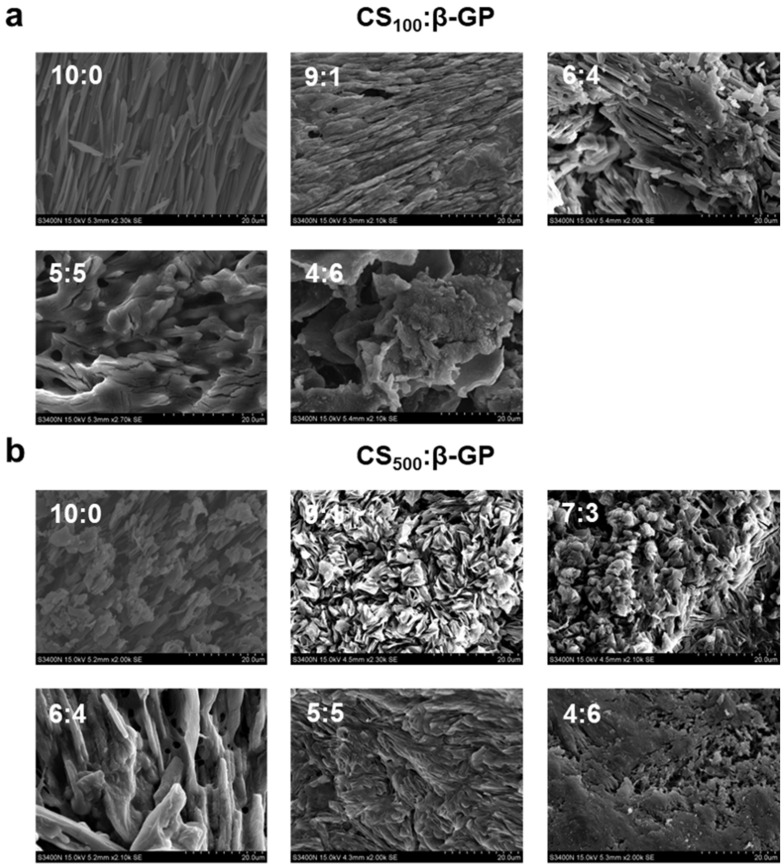
SEM of the non-gel group of CS_100_ (**a**) and CS_500_ (**b**). Scale bar: 20 μm.

The morphology and microstructure of each cryogel and freeze-dried composite can be seen in the SEM images. Cryogels and freeze-dried composites were obtained from original samples by freeze-drying, and the structures were related to the expected structure of the hydrogels and composites. As shown in Figure 5, each sample has a network structure distinguished by the size and degree of porosity, which is in agreement with published results [46]. Gel_100-73_ had large and loose pores with an overall elongated appearance (Figure 5a). The network structures of Gel_100-82_ and Gel_500-82_ were relatively dense, and their pores were small and nearly circular (Figure 5b,c). In contrast, the pores of Gel_500-82_ were slightly smaller than those of Gel_100-82_, which may be related to the larger molecular weight of CS_500_. The large molecular weight determines the relatively long molecular chain of CS_500_, leading to enhanced intermolecular interaction forces (hydrogen bonding and hydrophobic interactions). This is the same principle as that in a study in which the introduction of titanium (Ti) assisted in the enhancement of gel strength [47]. The SEM images of each composite (Figure 5d–f) presented similar results to the SEM images of the corresponding hydrogels, with little variation in the grid size of the gel organization; however, the grid structure of Gel_100-73_/NPs and Gel_100-82_/NPs was not as pronounced as that of the corresponding hydrogel samples due to the influence of the surface charge of the NPs. The microstructure of the group without gelation showed neatly arranged fibers, without obvious reticular structures or complex winding structures (Figure 6). This indicated that, in the group without gelation, the addition of β-GP formed a hydration layer around the CS molecules; thus, the molecular chains originally in the entangled state were extended and arranged in order, but no cross-linking occurred under the effect of temperature or no network structure was formed after cross-linking.

#### 2.2.5. Swelling Ratios

The swelling rate is commonly used to measure the degree of water absorption and swelling of hydrogels which, to a certain extent, determines the application field and specific use of hydrogels [48,49]. Gel_100-73_ and Gel_100-82_ reached the maximum swelling rate at around 50 min. Gel_500-82_ reached the maximum swelling rate at around 40 min. The swelling rate of the gel samples rapidly reached the maximum value within 1 h and then gradually stabilized (Figure 5g). Both Gel_100-82_ and Gel_500-82_ had a maximum swelling of more than twice the original mass, at 220.54 ± 6.20% and 248.55 ± 10.02%, respectively, indicating excellent swelling efficiency. The swelling rate of Gel_100-73_ was relatively small but also close to 100%. An excellent swelling effect indicates that the hydrogel has a strong water-holding capacity (WHC), which enables the hydrogel to provide a moist environment for the surrounding contacts. At the same time, correlating with the SEM images of the samples, it can be inferred that the WHC of the loosely structured reticular structure is not as good as that of the tightly structured reticular structure [45]. Similar to the previous results of Bhuiyan et al., the change in the CS ratio is also one of the factors affecting the swelling rate [43]. The increase in the CS ratio contributes to an increased swelling ratio, likely because the introduction of the polar groups generates a more hydrophilic chemical structure.

#### 2.2.6. In Vitro Release Assays

**Figure 7:**
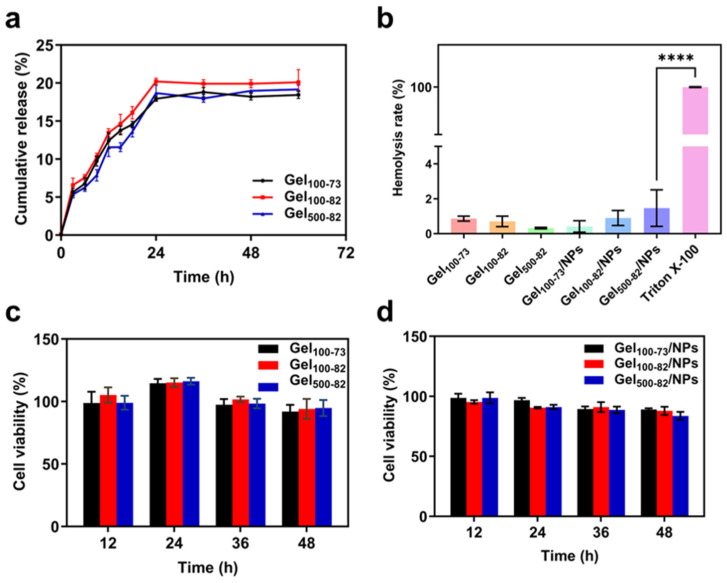
Characterization of nanoparticle–hydrogel composite system. (**a**) efficiency of nisin Z release from Gel_100-73_, Gel_100-82,_ and Gel_500-82_, (**b**) hemolysis rate of samples, (**c**) in vitro toxicity of gel samples, (**d**) in vitro toxicity of gel composite system. **** *p* < 0.0001 indicates significant differences between samples and Triton X-100 control group.

The composite system released nisin Z into the PBS solution it was in contact with, and the release efficiency was determined using the BCA method. The experimental results show that the nisin Z release rate of the sample reached the maximum within 24 h, after which the nisin Z was released steadily (Figure 7a). It is worth noting that the release rate of Gel_100-82_ was always higher than that of Gel_100-73_ and Gel_500-82_, finally reaching 20.09 ± 1.68%. The release rates of Gel_100-73_ and Gel_500-82_ ultimately stabilized at 18.43 ± 0.47% and 19.16 ± 0.57%, respectively. Compared with complexes reported in the literature, the release rates of our samples were low [50,51]. The method used in the release rate calculation was based on the entrapment efficiency of nanoparticles. However, some nisin Z may remain on the surface of the NPs and be bound by intermolecular forces, resulting in a high encapsulation efficiency. This part of nisin Z is also unable to be subsequently released from the gel tissue, thus resulting in a low overall release rate from the sample. The low level and sustained drug release efficiency are more suitable for use in situations where tight control of the drug concentration is required. The ophthalmic drug delivery system developed by Hsu et al. exploited this property [52].

#### 2.2.7. In Vitro Toxicity Assays

For the toxicity experiment, the CCK-8 method was used to explore the cytotoxicity of the samples. The cytotoxicity levels of materials are generally classified into four grades: grade 0 (no cytotoxicity, cell viability ≥ 100%), grade 1 (mild cytotoxicity, cell viability ranging from 75% to 99%), grade 2 (moderate cytotoxicity, cell viability ranging from 50% to 74%), and grade 3 (severe cytotoxicity, cell viability ranging from 25% to 49%) [50,51]. The cell viability of the hydrogel samples was more than 100% when they were in contact with the cells for 24 h (Figure 7c). With the extension of time, the cell survival rate decreased, but it was still grade 2 with a level of mild cytotoxicity. The cell viability of the composite systems showed a decreasing trend with the extension of time, which may be related to the introduction of antimicrobial peptides (Figure 7d). However, the overall level was still maintained at grade 2, showing great biocompatibility. Compared to the complex encapsulated with inorganic nanoparticles, our material presents lower cytotoxicity, correlated with the safety of nisin Z [53]. The cytotoxicity of previously developed composites utilized in wound dressings is equivalent to that of this product, which is a decisive factor of the material as a wound dressing [54,55].

#### 2.2.8. Hemolysis Evaluation

The hemolysis rate is an important factor in evaluating the biocompatibility of materials, which represents the degree of damage to erythrocytes. To a certain extent, the lower the hemolysis rate, the safer the material, as hemolysis will not occur when the blood is in contact with the material [56]. It can be seen from Figure 7b that the hemolysis rate of each gel sample and composite system was lower than 2%, and there was no significant difference between the samples. Previous materials typically used 5% as a critical point for the hemolysis rate; from this point of view, our materials have the safety and biocompatibility required for biological dressings [51]. In practical application, the effects of endogenous and exogenous factors on the hemolysis rate should be comprehensively considered. The ability of our material to provide a low hemolysis rate at the point of use enhances the tolerance to hemolysis caused by internal factors.

#### 2.2.9. In Vitro Antibacterial Characterization

**Figure 8:**
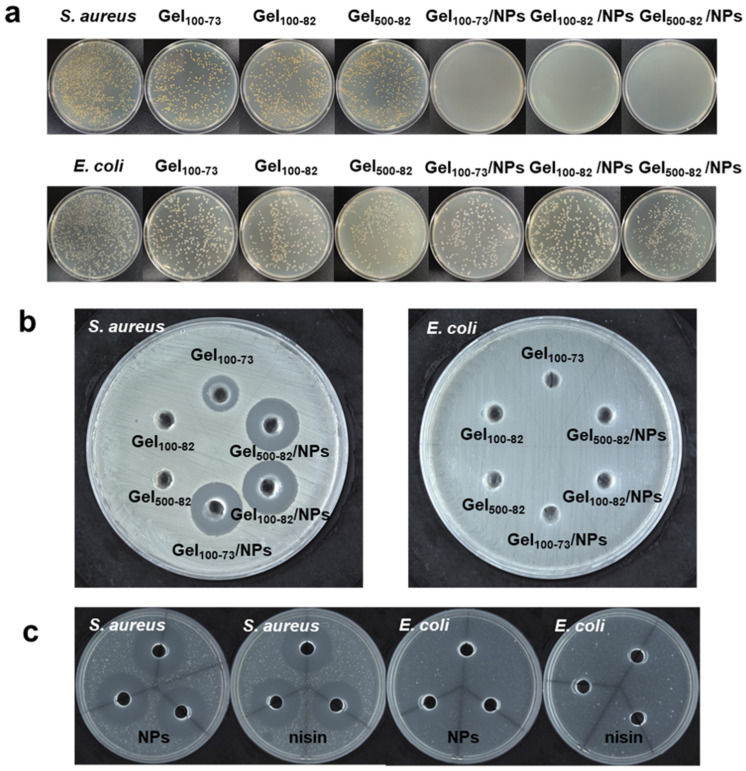
Antibacterial properties of the material. (**a**) results of plate count assay for the inhibition of samples against *S. aureus* and *E. coli*, (**b**) results of inhibition zone test of samples against *S. aureus* and *E. coli*, (**c**) Inhibition zone results of NPs and nisin Z against *S. aureus* and *E. coli*.

The antibacterial activity of the samples was evaluated using the inhibition zone and colony counting method. As shown in Figure 8b, neither the hydrogel nor the complex exhibited antibacterial properties against *E. coli*. According to previous studies, nisin Z is able to inhibit Gram-positive bacteria by forming membrane channels with them, leading to the efflux of cellular contents. However, nisin Z is usually unable to form membrane channels on the surface of Gram-negative bacteria and needs to disrupt the cellular membranes with the help of other active substances before exerting an inhibitory effect through the outer membranes [57,58,59]. Gel_100-73_ and each complex exhibited significant inhibitory effects against *S. aureus*, with the diameter of the zone of inhibition for each complex against *S. aureus* provided in Table S2. The results of the colony counting method also showed the same antibacterial effect (Figure 8a). There was no significant difference in the number of *E. coli* colonies on the serial dilution plates, but the inhibitory effect on *S. aureus* was more obvious at a 10^3^-fold dilution. In Figure 8c, the antibacterial effect of the nisin Z solution and NPs is also shown, and it can be observed that the antibacterial effect of the above complex mainly comes from nisin Z. The antibacterial properties of Gel_100-73_ may be related to those of the CS [60,61,62]. Furthermore, Gel_100-82_ and Gel_500-82_ showed similar dense reticulation structures in SEM results. Combined with this, we envisioned that the failure of both Gel_100-82_ and Gel_500-82_ to inhibit *S. aureus* could also be due to their overly dense structures, which blocked the inhibitory sites of chitosan in the hydrogels. *S. aureus* is a common foodborne pathogen that also poses a great risk in human wounds and disease infection. The complex shows an excellent inhibitory effect against *S. aureus*, and the loaded antimicrobial peptides can also avoid drug resistance, which has great application prospects in biology [63]. A recently reported nisin nanoparticle pre-formulation, with improved antibacterial effects encapsulated with a special material, has a similar effect to our material [64]. The nano-encapsulation technology significantly enhances the antibacterial efficacy of active substances, thereby ensuring their effectiveness and potentially even improving it. Another experiment utilizing nisin as a gel coating further substantiated its antibacterial efficacy [65]. This investigation not only validated the functionality of NPs but also provided evidence for the encapsulation and subsequent release of NPs via hydrogel.

## 3. Materials and Methods

### 3.1. Materials

For this study, the supplier of nisin (3%) was CHIHONBIO Co., Ltd. (Luoyang, China), and its specific species was nisin Z. γ-Polyglutamic acid (MW > 700 kDa) was provided by Shanghai Yongchuan Biotechnology Co., Ltd. (Shanghai, China). Chitosan and β-Glycerol phosphate disodium salt (β-GP, purity > 95%) were purchased from Shanghai Yongchuan Biotechnology Co., Ltd. (Shanghai, China). The molecular weights of CS were 100 (degree of deacetylation 85%) and 500 (degree of deacetylation 80%) kDa, denoted as CS_100_ and CS_500_, respectively. The hydrochloric acid (37%) used in the experiment was provided by the Hazardous Chemicals Management Department of Ningbo University, and substances, such as acetic acid glacial (99.5%), PBS buffer, and paraffin oil (99%) were also provided by Shanghai Yongchuan Biotechnology Co., Ltd. (Shanghai, China).

### 3.2. Synthesis of Nanoparticles

The synthesis of nanoparticles employed a modified approach based on previous methods [66]. After being dialyzed and freeze-dried for 24 h using a SCIENTZ-18N freeze-dryer, purchased from Ningbo Scientz Biotechnology Co., Ltd. (Ningbo, China), the purchased nisin Z samples were stored in a refrigerator at 4 °C for future use. For the synthesis of nanoparticles, the nisin Z aqueous solution was filtered through a disposable 0.22 μm membrane and then progressively added to the equivalent volume of a γ-PGA solution (4 mg/mL) and stirred for 30 min. CS_100_ was added to an acetic acid solution (1%, *v*/*v*) to form a 2 mg/mL chitosan acetate solution and agitated at room temperature until completely dissolved. The specific proportions of the various substances are detailed in Table S3. The mixed solution with nisin Z and γ-PGA was added dropwise to the acetic acid solution of CS_100_ and stirred overnight. It was then briefly homogenized using a handheld homogenizer and filtered through a disposable 0.22 μm membrane to obtain the NPs solution.

### 3.3. Zetasizer Particle Characterization

A Malvern Zetasizer Advance Series Lab (Malvern Panalytical, Malvern, UK) was used to measure the nanoparticle size, zeta potential, and polydispersity index (PDI). The nanoparticle size and polydispersity index were determined at 25 °C and a 173° scattering angle in a disposable cell (DTS0012) [67]. Similarly, potential measurements were also conducted in a specific sample dish (DTS1070). To ensure that the resultant values were within the confidence interval, the nanoparticle solution was diluted 5-fold using ultrapure water before measurement. The measurements were repeated three times for each sample.

### 3.4. Entrapment Efficiency of Nisin Z

Inspired by the prior approach of testing the encapsulation efficiency of gel beads, in this experiment, we permitted the unencapsulated nisin Z to enter the dialysate from the nanoparticle solution via dialysis and then utilized an Enhanced BCA Protein Assay Kit (Beyotime Biotechnology, Shanghai, China) to quantify the concentration of nisin Z in the dialysate [68]. The encapsulation rate of nisin Z was estimated using the following Equation (1). Dialysate of *n* times the volume of the nanoparticle solution was added to the dialysis vessel to facilitate calculation of the results. At the same time, we dried and weighed the NPs and calculated the loading capacity of each NPs sample for nisin Z according to the following Formula (2). The experiment was repeated to obtain three readings.

(1) $$Entrapment\;efficiency\;(\%)=\frac{Total\;nisin\;Z\;concentration-Unencapsulated\;nisin\;Z\;concentration}{Total\;nisin\;Z\;concentration}\times 100$$

(2) $$Loading\;capacity(\%)=\frac{Total\;mass\;of\;nisin\;Z-Unencapsulated\;nisin\;Z\;mass}{Total\;mass\;of\;NPs}\times 100$$

### 3.5. Preparation of Hydrogel and Nanoparticle–Hydrogel Composite System

We drew on previous ice bath methods for hydrogel fabrication [69] to identify the optimal ratios for achieving a temperature response hydrogel in subsequent experiments. A quantity of CS was weighed and added to a 0.1 mol/L hydrochloric acid solution with simultaneous magnetic stirring to prepare a hydrochloric acid solution with a CS concentration of 2% (*m*/*v*). It was mixed at room temperature until completely dissolved, then sterilized and stored in a refrigerator at 4 °C. An appropriate amount of β-GP was dissolved in sterile water to obtain a 45% (*m*/*v*) aqueous solution of β-GP, followed by its filtration through a 0.22 μm disposable sterile needle filter membrane. To create a clear and uniform CS/β-GP combination, the 45% aqueous β-GP solution was added dropwise to the 2% CS solution and stirred continuously in an ice bath for 30 min. The mixture was then incubated in a water bath for a set amount of time at a suitable temperature to form a hydrogel.

In preparing the composites, the CS/β-GP mixture was first stirred in an ice bath for 30 min according to the above steps. Next, the NPs solution was added drop by drop to the CS/β-GP mixture under ice bath conditions. To ensure uniform distribution of NPs, the mixture was again stirred in an ice bath for 30 min. Then, the nanoparticle–hydrogel composite system can be generated following further water bath incubation [70].

### 3.6. Determination of the Optimal Ratio of Gel Components

Two molecular weights (CS_100_ and CS_500_) of CS were selected, and the ratio of CS to β-GP was adjusted to investigate the optimal gel composition. Provided that the total volume was the same, the volume ratios chosen for the experiment were 10:0, 9:1, 8:2, 7:3, 6:4, 5:5, and 4:6. Preliminarily, the gel environment temperature was set at 37 °C, with a total testing duration of 15 min [71]. The group with the best gelation was selected visually using the inversion method, which involved inverting the container every 30 s for 15 min to observe the fluidity of each sample, determine whether gelation occurred, and document the time it took for gelation. In addition, the gelation state of the sieved groups at various temperatures was recorded to observe the transition from solution to gel, and temperature gradients were set up between 5 °C and 55 °C. The whole experiment was repeated three times.

### 3.7. Rheological Characterization

The dynamic oscillation tests were performed using a DHR-2 rheometer (Discovery Hybrid Rheometer, TA Instrument, Newcastle, DE, USA), including time scanning and oscillatory temperature change [72]. For the experiments, we equipped a parallel disk of 40 mm and lowered it to a gap of 1 mm. To prevent moisture loss, a ring of paraffin oil was dripped around the sample to seal it after the disc was lowered. Time scans were performed at 37 °C to record the dynamic modulus versus time over 1000 s, enabling the time required for gelation to be analyzed. Oscillatory temperature changes were performed to record the temperature at which the sol–gel transition behavior occurred, and the temperature was programmed to rise from 5 °C to 55 °C. A constant frequency of 1 Hz was set for all these measurements. The measurements were repeated three times for each sample.

### 3.8. Fourier Transform Infrared Spectroscopy (FTIR)

The hydrogel was freeze-dried for FTIR spectral analysis using a Nicolet iS50 FTIR spectrometer (Thermo Fisher Scientific Inc., Waltham, MA, USA). The freeze-dried samples were evenly mixed with KBr at a ratio of 1:100 and finely ground using a clean and dry agate mortar. The powder was placed in a tablet press and scanned in the energy range of 400 to 4000 cm^−1^, provided that the tablet was uniform and crack-free [73]. The measurements were repeated three times for each sample.

### 3.9. Scanning Electron Microscopy (SEM)

The morphological structures of the samples were observed using a Hitachi S-3400 scanning electron microscope (Hitachi High-Technologies Corporation, Tokyo, Japan). In order to photograph the internal structure of the hydrogel, the freeze-dried sample was cut into cubes of the same size to photograph the cutting section. The sample was loaded on a cylindrical stage with conductive tape and scanned at an accelerating voltage of 15 kV after spraying a layer of gold [74]. The experiment was performed three times in parallel.

### 3.10. Swelling Ratios

The swelling rate of the sample was determined using the weighing method; that is, the proportion of mass increase in the freeze-dried sample after water absorption over a certain period of time was determined. After weighing, the freeze-dried samples were soaked in distilled water at room temperature and subsequently taken out and weighed every hour (recording measurements every ten minutes for the first hour) after drying the surface moisture with filter paper [75]. The experiment was performed three times in parallel.

### 3.11. In Vitro Release Assays

The release efficiency of nisin Z in the composite system was monitored for 60 h at 37 °C. The sustained-release test was carried out in a glass bottle using 2 mL gel complex and 18 mL PBS solution. A certain amount of PBS solution was taken out regularly, and the content of nisin Z was determined using the BCA method. The corresponding volume of PBS was supplemented for subsequent measurements [52]. The measurements were repeated three times for each sample.

### 3.12. In Vitro Toxicity Assays

RAW264.7 macrophages were used for the CCK-8 (2-(2-methoxy-4-nitrophenyl)-3-(4-nitrophenyl)-5-(2,4-disulfophenyl)-2H-tetrazolium monosodium salt) method to evaluate the cytotoxicity of the materials [76]. The RAW264.7 cell line was stored at the Department of Food Science and Engineering, Ningbo University. After activation and passage of RAW cells, a batch of cells with good growth and a differentiation of less than 20% was selected for the experiment. The sample was co-cultured with the cell complete medium (the same volume) in a 6-well plate for 12 h to obtain the hydrogel extract. Cells were seeded into 96-well plates at a density of 5 × 10^4^ cells/well and incubated at 37 °C and 5% CO_2_ for 12 h. Subsequently, the medium was discarded, 100 μL of the extract was added to each well, and a control group with complete medium was set. After co-incubation for 12 h, 24 h, 36 h and 48 h, the original culture was discarded and 100 μL of complete culture medium containing 10% CCK-8 was added. Simultaneously, a control group with only CCK-8 and complete culture medium without cells was established. The culture plate was incubated for 1.5 h, and the absorbance at 450 nm was measured using a microplate reader. The experiment involved the conduction of three parallel tests. The cytotoxicity was calculated using the following formula:

(3) $$Cell\;viability\;(\%)=\frac{A_{s}-A_{b}}{A_{c}-A_{b}}\times 100$$

where A_s_ represents the absorbance of the experimental wells (containing cells, medium, CCK-8 and extract), A_b_ represents the absorbance of the blank wells (containing medium and CCK-8), and A_c_ refers to the absorbance of the control wells (containing cells, medium and CCK-8). The measurements were repeated three times for each sample.

### 3.13. Assay for Erythrocyte Hemolysis

The hemolysis assay was determined using the induction method, following a previous protocol with minor modifications [77]. Anticoagulated fresh sheep blood for testing was centrifuged at 2000 rpm and 4 °C for 10 min, and the supernatant was removed. Erythrocytes were resuspended in PBS and washed three times with centrifugation at 2000 rpm and 4 °C for 10 min. The concentration of the washed cells was adjusted to 1 × 10^8^ cells/mL (200 µL of erythrocyte precipitate was added to 10 mL of PBS via suction). Blood cell suspension (100 µL) was mixed with 100 µL of each sample extract in a centrifuge tube; sterile PBS and Triton were used as controls for 0% and 100% hemolysis, respectively. After exposing the mixture for 30 min in an incubator at 37 °C, the mixture was centrifuged at 2000 rpm and 4 °C for 3 min. The absorbance of the supernatant was measured at 540 nm, and the hemolysis rate was calculated using the following formula:

(4) $$Hemolysis\;rate\;(\%)=\frac{A_{x}-A_{0\%}}{A_{100\%}-A_{0\%}}\times 100$$

where A_x_ refers to the absorbance of experimental wells (extract group), A_0%_ denotes the absorbance of blank wells (PBS group), and A_100%_ denotes the absorbance of control wells (Triton group). The measurements were repeated three times for each sample.

### 3.14. In Vitro Antibacterial Assays

The inhibition circle and colony counting methods were used to investigate the antimicrobial properties of materials. Gram-positive bacteria (*S. aureus* ATCC 6538) and Gram-negative bacteria (*E. coli* ATCC 25922) were selected for antibacterial experiments. The specific operation of the inhibition zone method was as follows: first, 100 μL of the bacterial solution (*S. aureus* or *E. coli*, OD_600 nm_ = 0.4) was poured into the agar solid medium and mixed well. The plate was then poured and punched, and 200 μL of the sample was added to each hole, one by one. Finally, it was cultured in an incubator at 37 °C for 24 h, and then the diameter of the inhibition zone was measured [78].

The colony plate counting method is divided into three steps: co-culture, dilution coating, and colony counting [79]. Equal amounts of bacterial solution and samples were co-cultured in an incubator for 18 h, and the group with only bacterial solution was used as a control. After culturing, the bacteria on the surface of the sample were removed using ultrasonic treatment. The bacterial solution from each group was diluted and coated with a gradient dilution. Three dilution factors—10^2^, 10^3^, and 10^4^—were selected. After 24 h of continuous culturing, colony counting was performed. The samples used for bacteriostatic experiments were prepared under sterile conditions, and the sterility of the raw materials and instruments was ensured. The measurements were repeated three times for each sample.

### 3.15. Statistical Analysis

The statistical analysis was carried out in triplicate. All data were expressed as the mean values and standard deviations (SDs) (*n* = 3). The images were generated using GraphPad Prism 10 (GraphPad Software, San Diego, CA, USA) or Origin 2021 software (OriginLab Corporation, Northampton, MA, USA). Significant differences were defined as *p* < 0.05.

## 4. Conclusions

We embedded nisin Z in nanoparticles and then used temperature-sensitive hydrogel as a carrier to obtain an effective sustained-release nanoparticle–hydrogel composite system. To achieve efficient encapsulation and release, we optimized the design of both the NPs and hydrogel. Within the experimental range, the NPs with a nisin Z content of 5 mg/mL were ultimately designated for the highest encapsulation effectiveness (86.18%) and optimal particle properties. The thermosensitive hydrogel was fabricated via the electrostatic interaction between CS and β-GP, and the three optimal gel groups (Gel_100-73_, Gel_100-82_, and Gel_500-82_) were determined using the inverted tube method. The thermosensitive gel phenomenon is observed only when the interaction force between CS and β-GP is relatively balanced; otherwise, they will remain in a solution or sol state. The NPs–hydrogel composite system had a strong antibacterial effect on *S. aureus* and great biocompatibility, attributed to the efficient encapsulation and sustained release (20.09 ± 1.68%) of nisin Z. In this study, we attempted to incorporate nisin Z into a nanoparticle–hydrogel composite system, resulting in a novel encapsulation form for nisin Z in the biomedical field, particularly for trauma dressings.

## Data Availability

The data presented in this study are available on request from the corresponding author.

## Supplementary Materials

The following supporting information can be downloaded at: https://www.mdpi.com/article/10.3390/md22090428/s1, Table S1: Characterization results of all nanoparticles; Table S2: Circle of inhibition diameters of nisin Z, NPs and composites on *S. aureus*; Table S3: Specific compositions of hydrogels, NPs and composites.
