# Supplementary material for: Synthesis and Characterization of a Novel Chitosan-Based Nanoparticle–Hydrogel Composite System Promising for Skin Wound Drug Delivery
Source: Mar Drugs. 2024 Sep 21;22(9):428. doi: 10.3390/md22090428 (PMC11433214; doi:10.3390/md22090428)
Supplement: Supplementary file 1 [file marinedrugs-22-00428-s001.zip › marinedrugs-3189540-supplementary.pdf]

Supplementary materials

Table S1. Characterization results of all nanoparticles.

Nanoparticle samples (mg/mL)	Average hydrodynamic diameter (nm)	Zeta potential (mV)	PDI
3	136.48±3.58	26.69±0.53	0.535±0.069
4	119.56±4.24	24.51±0.45	0.626±0.015
5	122.72±4.88	23.89±0.37	0.473±0.063
10	-	28.96±0.78	> 0.7
15	-	30.7±0.36	> 0.7
20	-	36.93±0.53	> 0.7

Table S2. Circle of inhibition diameters of nisin Z, NPC and composites on *S. aureus*.

	S. aureus	Nisin Z	NPC	Gel_{10-73}/NPC	Gel_{10-82}/NPC	Gel_{50-82}/NPC
Circle of inhibition diameters (mm)	0.00	20.20±0.58	19.70±0.27	16.59±0.83	17.06±0.83	16.91±0.72

Table S3. Volume percentage of each component of hydrogels, NPs and composites.

	Gel_{100-73}	Gel_{100-82}	Gel_{500-82}	5 mg/mL NPs
CS_{100} (2%, m/v)	1.4	1.6	-	0.1
CS_{500} (2%, m/v)	-	-	1.6	-
HCl (1mol/L)	68.5	78.3	78.3	-
β-GP (45%, m/v)	13.5	9	9	-
H_2O	16.5	11	11	98.8
Nisin (20mg/mL)	-	-	-	0.5
γ-PGA (4mg/mL)	-	-	-	0.1
Acetic acid (1%, v/v)	-	-	-	0.5
